# Puerarin improves skeletal muscle strength by regulating gut microbiota in young adult rats

**DOI:** 10.1016/j.jot.2022.08.009

**Published:** 2022-09-21

**Authors:** Wenyao Yang, Bimin Gao, Ling Qin, Xinluan Wang

**Affiliations:** a Translational Medicine R&D Center, Institute of Biomedical and Health Engineering, Shenzhen Institutes of Advanced Technology, Chinese Academy of Sciences, Shenzhen, China; b Musculoskeletal Research Laboratory, Department of Orthopaedics & Traumatology, The Chinese University of Hong Kong, Hong Kong, China; c Joint Laboratory of Chinese Academic of Science and Hong Kong for Biomaterials of the Chinese University of Hong Kong, Shenzhen, China

**Keywords:** Puerarin, Skeletal muscle, Gut microbiota, SCFAs, ATP, adenosine triphosphate, CSA, cross-sectional area, CMC-Na, sodium carboxymethyl cellulose, EDL, extensor digitorum longus, GC–MS, gas chromatography-mass spectrometry, SCFAs, short-chain fatty acids, SOL, soleus

## Abstract

**Background:**

Sarcopenia is an age-related skeletal muscle dysfunction syndrome that is lacking validated treatments. Maximizing muscle strength in young adulthood may be a promising way to prevent sarcopenia in the elderly. The phytomolecule puerarin has been extensively used in clinical practice and reported to increase energy metabolism in skeletal muscle by directly targeting the skeletal muscle fiber. However, the bioavailability of puerarin is very poor, and almost 93% of puerarin stays in the intestine until excretion. Therefore, we hypothesize that puerarin may regulate gut microbiota to improve skeletal muscle strength and/or mass in adults.

**Methods:**

Twenty three-month old male Sprague Dawley rats were divided into two groups according to average weights, puerarin group (puerarin dissolved in 0.5% CMC-Na, 150 ​mg/kg/day, N ​= ​10), and control group (equal volume 0.5% CMC-Na, N ​= ​10). The treatment lasted for 8 weeks. Muscle weight, muscle fiber types and cross-sectional area (CSA), *ex vivo* muscle contraction test and grip strength were measured. 16S rDNA sequencing was employed to evaluate the gut microbiota composition in the sample of cecal content. Short-chain fatty acids (SCFAs) in cecal and serum were analyzed by gas chromatography-mass spectrometry. Adenosine triphosphate (ATP) concentration in skeletal muscle was also detected. Pearson's correlation was used to analyze the relations between SCFAs, ATP concentration and muscle function.

**Results:**

After puerarin treatment, grip strength, the specific twitch force, and the tetanic forces in the soleus (SOL) and extensor digitorum longus (EDL) muscle were significantly higher than those of the control group. The percentage and CSA of type II muscle fiber in EDL was higher in the puerarin group than those in the control group. Puerarin treatment significantly changed the gut microbial constitutes. Two SCFAs-productive microbiota, the families Peptococcaceae and Closteridiales, were significantly higher in the puerarin group than those in the control group, while the ratio of Prevotellaceae/Bacteroidaceae (P/B), a muscle atrophy indicator, was lower in the puerarin group. As expected, there were significant linear correlations between the concentrations of SCFAs, including cecal total SCFAs, serum *n*-butyric acid and total SCFAs, and skeletal muscle strength and function, including the twitch force and tetanic force of SOL and EDL, as well as the forelimb grip strength.

**Conclusion:**

In conclusion, puerarin improved the forelimb grip strength and muscle contraction function in young adult rats. The underlying mechanism may include that puerarin increased SCFAs production by regulating gut microbiota, augmented ATP synthesis and skeletal muscle strength.

***The translational potential of this article***: Our study finds that a clinical used phytomolecule puerarin has the potential of improving skeletal muscle strength in young adult rats. As puerarin has long-term clinical experience and shows good safety, it might be a potential candidate for developing muscle strengthening agents.

## 1 Introduction

Skeletal muscle makes up 40% of total body weight in adults [1]. Muscle mass and strength peak in early adulthood, followed by a more substantial decline from the fifth decade onwards, culminating in the drastic loss of muscle strength characteristic of sarcopenia in later life [2,3]. Sarcopenia is an age-related skeletal mass wasting syndrome and is associated with a marked increase in morbidity and mortality [4,5]. In recent years, despite the development of great efforts to enhance muscle mass and strength against sarcopenia in the elderly, to date, there are no approved drug therapies for sarcopenia [[6], [7], [8]]. In fact, there is a life course approach to sarcopenia, which suggests that muscle mass and function in older people depend not only on the rate of functional decline in later life but also on the functional peak reached in young adulthood [9]. Therefore, the European Working Group on Sarcopenia in Older People recommendations for enhancing muscle mass and strength in young adulthood might be a promising strategy to prevent or delay sarcopenia in later life [3,9].

Puerarin (daidzein 8-C-glucoside) is an isoflavonoid from the root of the *Puerariae lobate* (Willd) Ohwi [10], which has been extensively used as a food supplement or medicine in China [11]. Clinically, injection, tablet, and capsule forms of puerarin are available for treatment of cardiovascular and cerebrovascular diseases [12,13]. Recently, puerarin was reported to exert beneficial effects on musculoskeletal diseases, such as osteoporosis [14] and muscle wasting in diabetes [15]. A recent study showed that puerarin ameliorated skeletal muscle wasting in diabetic mice, directly by targeting mitochondrial function in L6 myotubes [16]. However, the absolute bioavailability of puerarin is only approximately 7% [17], which means almost 93% of puerarin stays in the intestine until excretion. Recently, Li et al. found that puerarin modulated the gut microbiota disorder in ovariectomized (OVX) rats [18], providing the potential mechanism.

The gut microbiota constitutes the community of microbes present within the gastrointestinal tract. This microbial ecosystem plays an important role in the host, facilitating the metabolism of dietary nutrients, synthesizing micronutrients and cofactors, modulating the mucosal immune system, and influencing energy balance [19]. Short-chain fatty acids (SCFAs), as metabolites mainly produced by gut microbiota, are important substrates of gluconeogenesis and participate in the regulation of central metabolism [20]. Moreover, SCFAs trigger host signals by inhibiting histone deacetylase (HDAC) [21] or activating G protein-coupled receptors (such as GPR41 and GPR43), which are activated to regulate the release of glucagon-like peptide 1 (GLP-1) and neuropeptide Y. GLP-1 ameliorated muscle wasting by suppressing muscle atrophic factors and enhancing myogenic factors [22]. Neuropeptide Y and its receptors expressed in skeletal muscle regulated mitochondrial function [23]. SCFAs are widely thought to mediate the relationship between the gut microbiota and skeletal muscle at least partly [24]. Germ-free mice do not produce SCFAs in the gut due to the lack of fermenting microbes [25]. It was found that conventional mice harvest more energy from the diet and are heavier than GF mice [26]. The growing evidence has supported the existing of gut–muscle axis [24,27,28]. Therefore, we hypothesized that puerarin might improve skeletal muscle strength *via* regulating the gut–muscle axis, an indirect mechanism on muscle tissue.

In this study, we investigated the effects of puerarin on the physical performance and skeletal muscle strength in young (3-month-old) adult rats. The 16S rDNA sequencing was employed to evaluate the gut microbiota composition in the sample of cecal content and SCFAs were analyzed to reveal the underlying indirect-targeting-muscle mechanism of puerarin in the intestine.

## 2 Materials and methods

### 2.1 Animal model

Twenty three-month old male Sprague Dawley rats (525–575g) were used in the experiment. They were randomly divided into two groups. Ten rats were given puerarin (Natural Field Bio-technique, Xian, China) dissolved in 0.5% sodium carboxymethyl cellulose (CMC-Na) by gavage for 8 weeks (150 ​mg/kg/day) as puerarin group, and others were given the same volume of 0.5% CMC-Na as the control group. The research protocol was approved by the animal experimentation ethics committee of the Shenzhen Institute of Advanced Technology, Chinese Academy of Sciences (Reference No. SIAT-IRB-170305-YGS-WXL-A0314).

At the endpoint, the muscle of extensor digitorum longus (EDL) and soleus (SOL) [29] of the right side was carefully isolated, which was then put in the tissue bath with mammalian Ringer's solution for *ex vivo* contraction test. The contralateral muscle was harvested after being weighed, snap-frozen with optimum cutting temperature compound (O.C.T, SAKURA, USA) in liquid nitrogen for 20s, and stored at −80 ​°C for histological, immunofluorescence examination and qPCR.

### 2.2 Grip strength measurement

Forepaws grip strength of the rat was measured with a force gauge (BIO-GS3, BIOSEB, FL, USA). Rats held by the tail grasped the grid connected to the force gauge with their forepaws [30]. The tails of rats were pulled slowly until the rat released their forepaws from the grid. The peak force of each test was recorded, and three repeats were collected and averaged.

### 2.3 Ex vivo contraction test

*Ex vivo* muscle contraction test was conducted according to our established protocols using an *ex vivo* muscle functional test system (1305A, Aurora Scientific Inc., Newmarket, Canada). At designated time points the rats were incised under general anesthesia, and SOL and EDL muscles of the right hind limb were freshly isolated carefully together with Achilles tendon for functional assessment. The threads were tied around the ends of the Achilles tendon to immobilize the muscles and keep them straight. After 15 ​min stabilization, the muscle was activated by twice tetanic contractions (1A, 300 ​ms, 100 ​Hz) at 5 ​min intervals. The optimal length (L0) of the muscle was determined by continuous stimulations with increasing muscle length until the new response value was equal to the former one. The tetanic force in SOL and EDL was detected by stimulation at 80 ​Hz for 800 ​ms and 100 ​Hz for 500 ​ms, respectively. Three tetanic responses were measured at 5 ​min intervals and averaged as the tetanic force (Ft). After the measurements, the EDL and SOL were dried and weighed. The EDL and SOL from the left leg were also isolated and weighted. Muscle mass was the average for both legs. Muscle cross-sectional area (CSA) and specific tetanic force (SFt) were calculated as previously described [31].

### 2.4 Myosin ATPase staining

Muscle samples collected from the SOL and EDL were cryo-sectioned for histological examination. Muscle samples sectioned at 8 ​μm thick (CM1950, Leica, Germany) were mounted on silane-coated glass slides. Muscle fibers characteristics of SOL and EDL muscles were analyzed using myosin ATPase staining [32] at pH 10.4 (DE0048, Leagene Biotechnology, Peking, China).

### 2.5 16S rDNA sequencing and bioinformatics analysis

Cecal content samples (N ​= ​5 each group) were collected in sterile tubes and stored at −80 ​°C. The sample DNA was extracted using MagPure Stool DNA KF kit B (Magen, China), and the quality was checked by running an aliquot on 1% agarose gel. Gut bacterial composition in two groups was analyzed *via* 16S rDNA genes analysis. Variable regions V3–V4 of bacterial 16S rDNA gene were amplified with degenerate PCR primers, 341F (5′-ACTCCTACGGGAGGCAGCAG-3′) and 806R (5′- GGACTACHVGGGTWTCTAAT-3′), to construct libraries. Alpha and beta diversity were estimated by MOTHUR (v1.31.2) [33] and QIIME (v1.8.0) [34] at the OTU level, respectively. The sample cluster was conducted by QIIME (v1.8.0) based on UPGMA. Kyoto Encyclopedia of Genes and Genomes (KEGG) and Clusters of Orthologs Groups (COG) functions were predicted using the Phylogenetic Investigation of Communities by Reconstruction of Unobserved States (PICRUSt) software [35]. Barplot and heatmap of different classification levels were plotted with R package v3.4.1 and R package “gplots”, respectively.

### 2.6 SCFAs determination in cecal contents and serum

SCFAs (acetic acid, propionic acid, butyric acid, isobutyric acid, valeric acid, and isovaleric acid) in the cecal contents (N ​= ​5 each group) were analyzed in gas chromatograph-mass spectrometry (GC–MS) by Qingdao Yixin detection technology service (Shandong, China) as described previously [36]. Before GC–MS analysis, the appropriate amount of cecal contents or serum were mixed with 5 times volume of water (phosphoric acid: ddH2O ​= ​1:3) by vortexing and centrifuging. Using 1 ​mL of ether each time and extract twice, then the extract was combined and volatilized to less than 1 ​mL for analysis. The filtrate was injected into a GC (Thermo GCMS ISQ LT). The SCFAs were separated by a fused silica capillary column (30m ​× ​0.25 ​mm ​× ​0.25 ​μm, TG WAX, Thermo Fisher Scientific Inc., USA). We carried out a complete methodological evaluation. The standard curves of SCFAs were obtained. Combined with the retention time and peak shape, the peak area of the target was quantified.

### 2.7 ATP measurement of muscle

Intracellular ATP levels were measured using a commercially available intracellular ATP measurement kit (Beyotime Biotechnology, China) according to the manufacturer's instructions [37]. Briefly, the frozen tissues were lysed with a lysis buffer and then centrifuged at 12,000 ​g for 10 ​min at 4 ​°C. After that, an aliquot of the supernatant plus an ATP detection solution was added to a 96-well plate (Corning, NY, U.S.A.). Luminescence was detected using a Synergy4 (Bio-Tek, Vermont, U.S.A). The ATP level was presented as nanomoles per milligram of protein (nM/mg protein).

### 2.8 Quantitative real-time PCR

Real-time PCR for quantification of RNA was carried out using a SYBR protocol on the fluorescence temperature cycler (Light cycler 96, Roche Molecular Diagnostics, Indianapolis, IN, USA). Real-time reactions were carried out in duplicate, and amplicons were analyzed by generating melting curves with continuous measurement of fluorescence. External standard curves were generated by amplification of 10-fold dilutions of GAPDH and the product of interest. Results were calculated as relative differences in target threshold cycle (Ct) values normalized to GAPDH. A list of primer sequences and expected product size is shown in Table S1.

### 2.9 Data and statistical analysis

Data are shown as the means ​± ​SEM. Graphpad Prism software 6.0 version (GraphPad Software, San Diego, California, USA) was used for illustrations, mapping, and statistical analysis. Wilcoxon and T-test analysis were used to compare the difference between two groups. All data analysis on 16S rDNA sequencing and bioinformatics analysis was used the Wilcoxon analysis, while others were analyzed by T-test. *P* ​< ​0.05 was considered as significant difference between puerarin and control group. Pearson's correlation was used to analyze the relations between SCFAs and ATP concentration and skeletal muscle contraction force, as well as forelimb grip strength. Coefficient of determination R^2^ ​> ​0.3 and R^2^ ​> ​0.5 means moderate and strong correlation, respectively [38]. *P* ​< ​0.05 indicate the significant correlation between two different variables.

## 3 Results

### 3.1 Puerarin enhances grip strength in rats

**Fig. 1:**
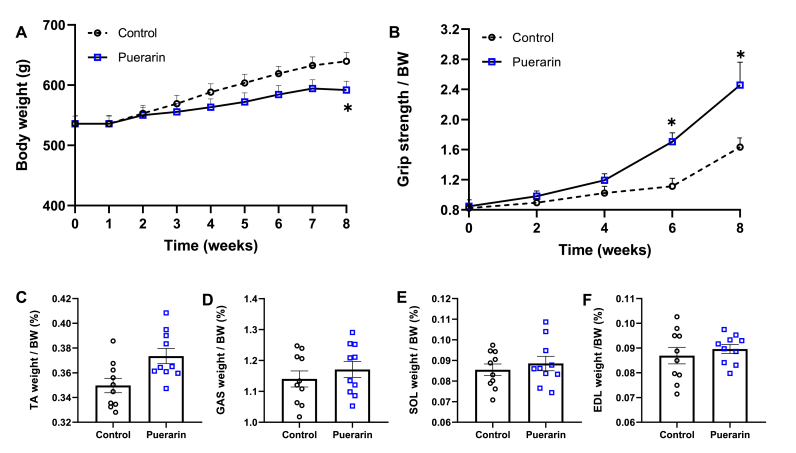
Puerarin enhances grip strength in rats. A: The body weights were measured every week. B: Changes in grip strength were detected every two weeks. C–F: Percentage of TA weight (C), GAS weight (D), SOL weight (E), and EDL weight (F) per body weight (BW). Data presented are individual values with means ​± ​SEM, N ​= ​10. T-test was used to evaluate differences between the control and the puerarin groups, ∗*P* ​< ​0.05.

After treatment for 8 weeks, the average body weight in the puerarin group was lower than that in the control group (Fig. 1A). Grip strength is a good marker for measuring physical performance [39]. The grip strengths per body weight were significantly (*P* ​< ​0.05) higher in puerarin group than those in the control group (Fig. 1B) at 6 and 8 weeks. There were no significant differences in the percentage of skeletal muscle mass in body weight, including the tibialis anterior (TA), the gastrocnemius (GAS), SOL and EDL muscles between two groups (Fig. 1C–F).

### 3.2 Puerarin improves skeletal muscle contraction force

**Fig. 2:**
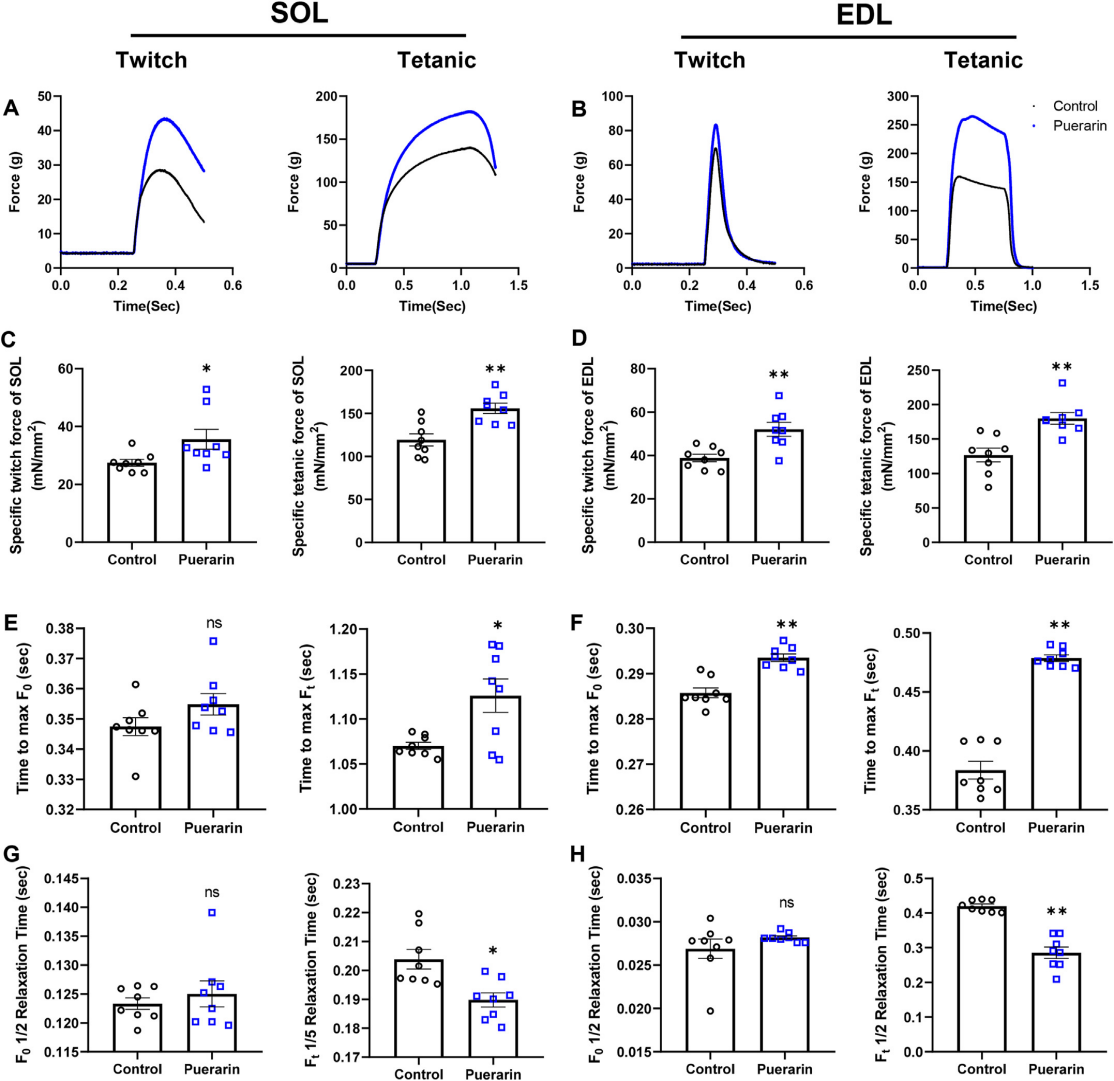
Puerarin increases skeletal muscle contraction force in rats. A&B: Representative force–time graphs during twitch and tetanic stimulations of SOL (A) and EDL (B). C: Specific twitch and tetanic force of SOL. D: Specific twitch and tetanic force of EDL. E&F: Time to the max force of twitch and tetanic in SOL (E) and EDL (F). G&H: Relaxation time of twitch and tetanic in SOL (G) and EDL (H). Data presented are individual values with means ​± ​SEM, N ​= ​8. T-test was used to evaluate differences between the control and the puerarin groups, ∗*P* ​< ​0.05, and ∗∗*P* ​< ​0.01.

We explored the effect of puerarin on single skeletal muscle function by testing muscle contractibility, and relaxation parameters under twitch and tetanic stimulation in SOL (Fig. 2A) and EDL muscles (Fig. 2B). In the SOL, the specific twitch and tetanic forces in the puerarin group were 29.6% and 30.6% higher than those in the control group, respectively (*P* ​< ​0.05 Fig. 2C). Similarly, puerarin increased the specific twitch and tetanic forces of EDL muscle by 34.3% and 41.2%, respectively, compared the control group (*P* ​< ​0.01, Fig. 2D). The time to max-tetanic force in SOL and EDL was 5.2% and 24.8% longer in the puerarin vs control group, respectively. Also, compared to the control group, the time to max-twitch force in EDL was 2.7% longer in puerarin group (*P* ​< ​0.05, Fig. 2E&F). The half relaxation time of tetanic force was reduced by 29.7% after treated with puerarin in the EDL (*P* ​< ​0.01, Fig. 2H) and a similar result was shown in the SOL (*P* ​< ​0.05, Fig. 2G).

### 3.3 Puerarin increases the percentage of fast fibers in fast-twitch muscle

**Fig. 3:**
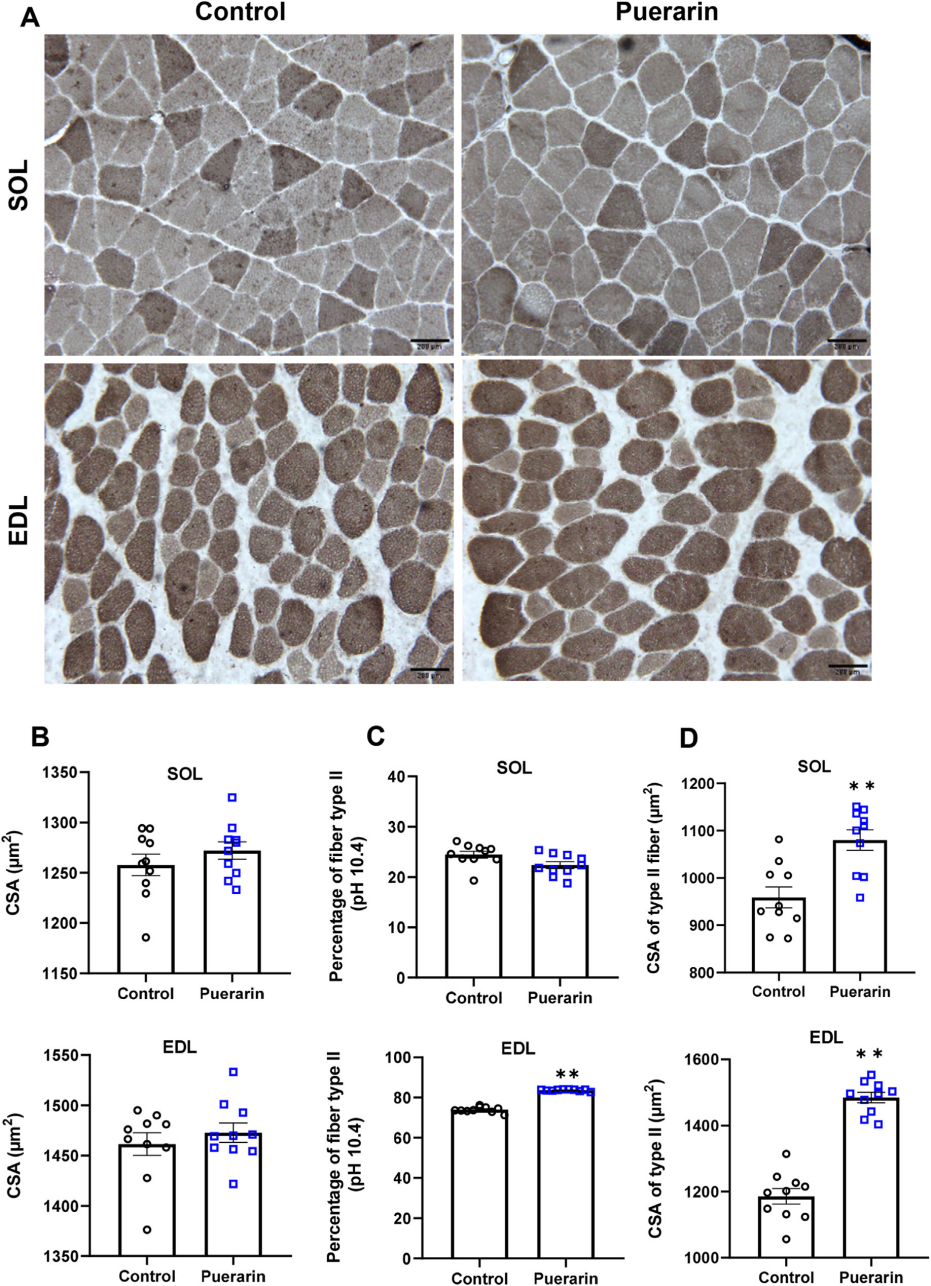
Puerarin increases the percentage of fast fibers in fast-twitch muscle. A: Frozen sections of SOL and EDL muscle samples were subject to myosin ATPase (pH ​= ​10.4) staining. B: The average CSAs of SOL and EDL muscles. C: Muscle fiber number ratios (type II/total fibers) of ATPase staining in SOL and EDL. D: The average CSAs of fiber type II in SOL and EDL. The values shown are the means ​± ​SEM, N ​= ​10. T-test was used to evaluate differences between the control and the puerarin groups, ∗∗*P* ​< ​0.01.

We investigated the effect of puerarin on the changes in skeletal muscle fiber areas and types (Fig. 3A). The CSAs of SOL and EDL were not significantly different between the two groups (Fig. 3B). However, there was a transform from an oxidative type (slow or type I fibers) to glycolytic type (fast or type II fibers) in EDL muscle in puerarin-treated rats, and the percentage of type II fibers in the puerarin group increased by 13.1% compared with that in control group (*P* ​< ​0.01, Fig. 3C). The CSAs of type II muscle fiber of SOL and EDL in the puerarin group were 12.7% and 25.2% higher than those in the control group, respectively (*P* ​< ​0.01, Fig. 3D).

### 3.4 Puerarin regulates the distribution of gut microbiota

**Fig. 4:**
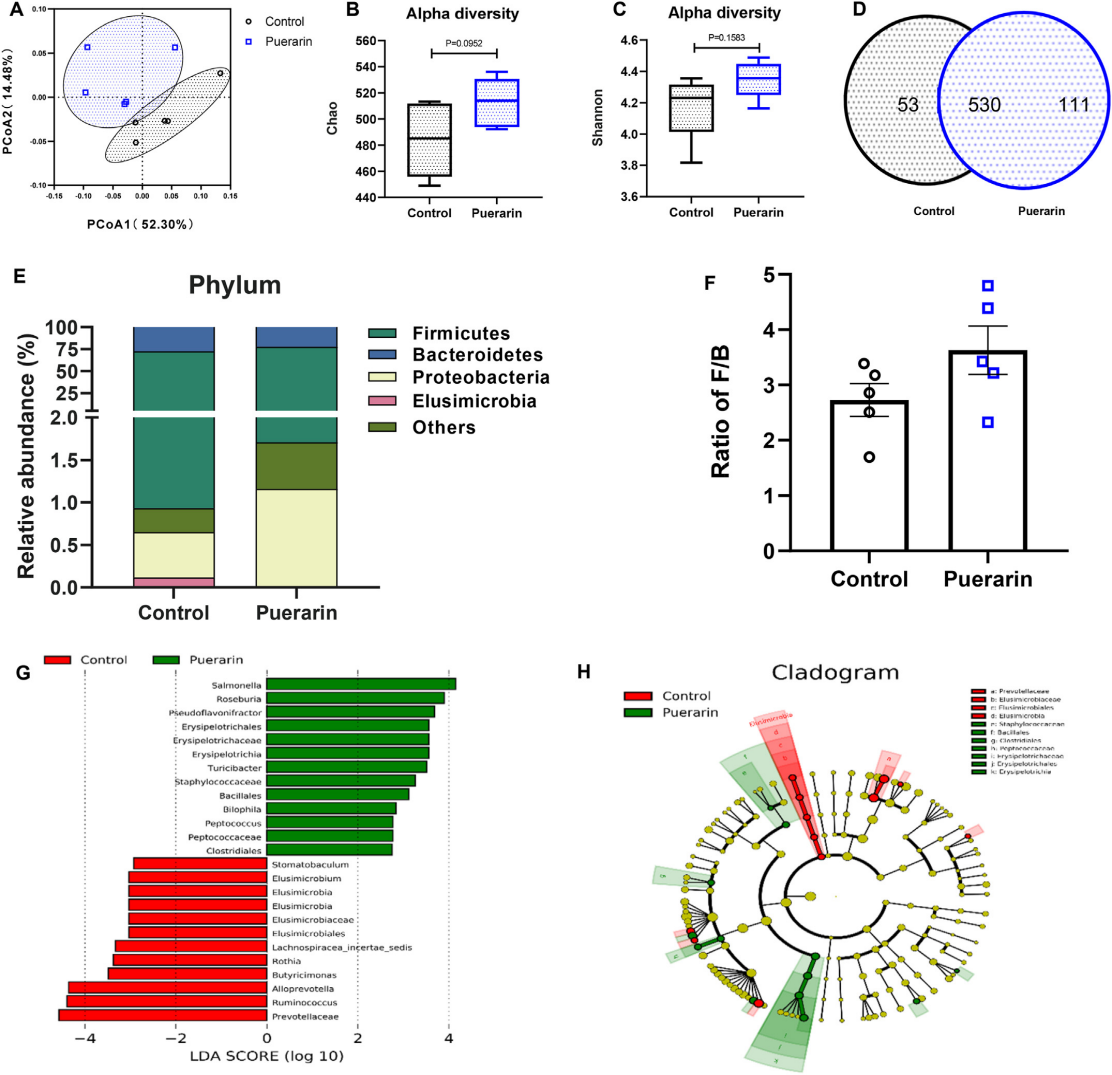
Effect of puerarin on gut microbiota in rats. A: Principal coordinates analysis (PCoA). B&C: Alpha diversity box chart of Chao1 index (B) and Shannon index (C). D: OTUs statistics-Venn chart: different color graphics in the figure represent the OTUs statistics of the two groups of rats, and the number of overlapping parts is the number of OTUs shared between the two groups. E: Average composition of the phylum level in two groups. Species with unannotated taxonomic levels and an abundance of less than 0.5% in the sample are merged into Others. F: The ratio of Firmicutes and Bacteroidetes. G: LEFSe analysis to show the species with significantly different in abundance between control and puerarin group. The threshold on the logarithmic LDA score for the discriminative feature is ​> ​2.0. H: Circular cladograms depicting the LEFSe results in two groups. The species with red bar present a significant increase in the control group and the species with green bar represent a significant increase in the puerarin group. Values shown are the means ​± ​SEM, N ​= ​5. Wilcoxon was used to evaluate differences between the control and the puerarin groups in Fig. B and C. T-test was used to evaluate differences between the control and the puerarin groups in Fig. F. (For interpretation of the references to colour in this figure legend, the reader is referred to the Web version of this article.)

Gut microbes influence skeletal muscle strength and physical performance in animals [40]. To describe the effect of puerarin in distribution of gut microbiota, we conducted principal co-ordinates analysis (PCoA) based on the weighted unifrac distance and found that gut microbiota constitutes were significantly different between the two groups (Fig. 4A). There were no significant differences for Alpha diversity index, Chao1 index (Fig. 4B) and Shannon index (Fig. 4C), between groups. The operational taxonomic units (OTUs) information was counted by the Venn diagram. Two groups have common 530 OTUs in the gut microbiota. There were 111 unique OTUs in the puerarin group and 53 unique OTUs in the control group (Fig. 4D).

To profile the detailed changes in gut microbiota, the relative abundance of flora at different levels was analyzed (Table S2-S4). All the flora whose relative abundances were more than 0.5% were identified. At the phylum level, Firmicutes and Bacteroidetes were the two dominant gut bacteria in both groups (Fig. 4E and Table S2). The ratio of Firmicutes (F) to Bacteroidetes (B) wasn't different between groups (*P* ​= ​0.126) (Fig. 4F). The threshold on the logarithmic LDA score for discriminative features was set to 2.0 [41]. LEFSe analysis (Fig. 4G) identified 13 bacteria in the puerarin group and 12 bacteria in the control group as potential microbial markers compared to each other. The circular cladograms (Fig. 4H) showed these different microbiomes in the phylogenetic tree.

**Fig. 5:**
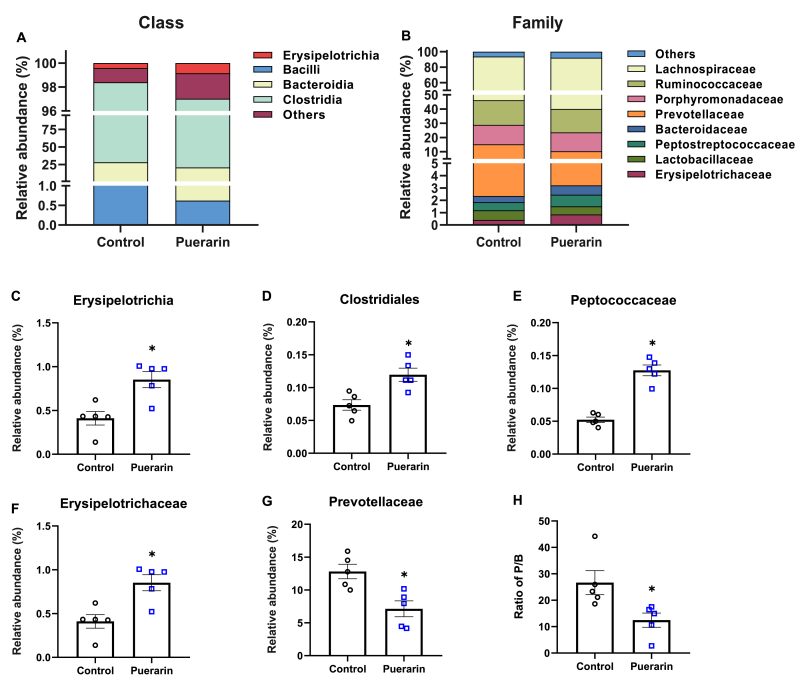
Effects of puerarin on class and family level taxonomic distributions of the microbial communities in cecal content. A&B: Average composition of the class (A) and family (B) level in two groups. Species with unannotated taxonomic levels and abundance of less than 0.5% in the sample are merged into Others. C–G: The relative abundance of Erysipelotrichia (C), Clostridiales (D), Peptococcaceae (E), Erysipelotrichaceae (F), and Prevotellaceae (G). H: The ratio of Prevotellaceae/Bacteroidaceae. Values shown are the means ​± ​SEM, N ​= ​5. Wilcoxon was used to evaluate differences between the control and puerarin groups for Fig. A–G, and T-test was used to evaluate differences between the control and the puerarin groups for Fig. H, ∗*P* ​< ​0.05.

Then we further analyzed the relative abundances of these microbiomes at the class (Fig. 5A and Table S3) and family levels (Fig. 5B and Table S4). The relative abundances of the most abundant class Clostridia were not different between groups (Table S3). The relative abundances of the class Erysipelotrichia were higher (*P* ​< ​0.05) in the puerarin group compared to the control group (Fig. 5C). At the family level, the gut microbiota of both groups was dominated by Lachnospiraceae, Ruminococcaceae, Porphyromonadaceae, Prevotellaceae, and Bacteroidaceae, whose relative abundances of gut microbiota were over 10% (Fig. 5B). Two families from the class Clostridia, Clostridiales and Peptococcaceae in the puerarin group were 62.8% (*P* ​< ​0.05) and 143.6% higher (*P* ​< ​0.05) than those in the control group, respectively (Fig. 5D&E). The relative abundance of the family Erysipelotrichaceae was 107.9% higher (*P* ​< ​0.05) in the puerarin group compared to the control group (Fig. 5F). The relative abundance of the family Prevotellaceae and the ratio of Prevotellaceae (P)/Bacteroidaceae (B) were lower by 44.3% (*P* ​< ​0.05) and 53.3% (*P* ​< ​0.05) in the puerarin group than those in the control group, respectively (Fig. 5G&H). Collectively, these results showed that treatment of puerarin for 8 weeks modulated the gut microbiota in young adult rats.

### 3.5 Prediction of potential metabolic functions of gut microbiota in rats

**Fig. 6:**
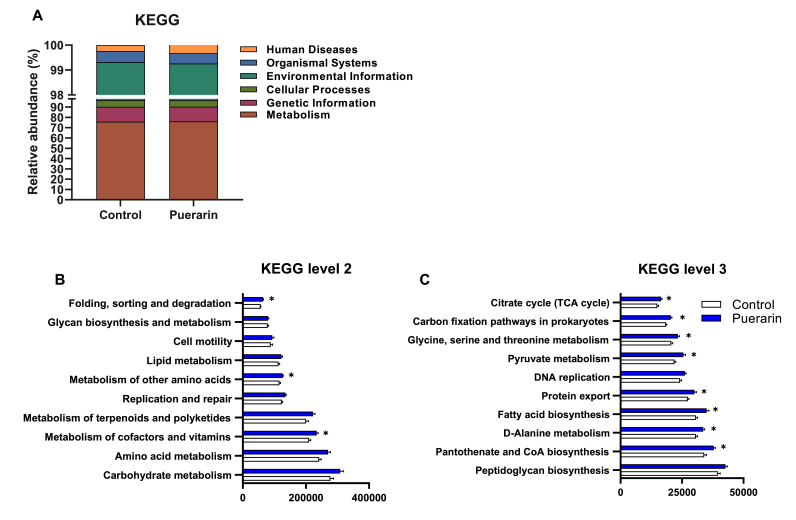
Prediction of gut microbiota function by KGEE. A: Relative abundance of biological entities and characteristics. B: KEGG function level 2. C: KEGG function level 3. Values shown are the means ​± ​SEM, N ​= ​5. Wilcoxon was used to evaluate differences between the control and puerarin groups, ∗*P* ​< ​0.05.

The KEGG database in PICRUSt2 software was used to predict the function of the flora. Based on the hierarchy of the protein network, KEGG pathway is classified into 3 levels. Here we showed six categories in the top level (metabolism, genetic information, cellular processes, environmental information, organismal systems, and human diseases) and top ten enriched subcategories in the second level and third level (Fig. 6A–C and Table S5-7). At Level 1, the biological functions of gut microbiota in both groups were mainly enriched in the metabolism pathway (Fig. 6A and Table S5). At Level 2, other amino acid metabolism, metabolism of cofactor and vitamin metabolism pathway, as well as protein folding, sorting, and degradation pathway were significantly higher in the puerarin group than those in the control group (*P* ​< ​0.05) (Fig. 6B and Table S6). Further analysis of KEGG level 3 revealed that many other pathways, such as those associated with pantothenate and CoA biosynthesis, fatty acid biosynthesis, pyruvate metabolism, glycine, serine, and threonine metabolism, and citrate cycle (TCA cycle), associated with ATP and SCFAs biosynthesis [42], were significantly higher in the puerarin group than those in the control group (*P* ​< ​0.05) (Fig. 6C and Table S7).

### 3.6 Puerarin improves the concentrations of SCFA in serum and cecal contents, and augments ATP in skeletal muscle

**Fig. 7:**
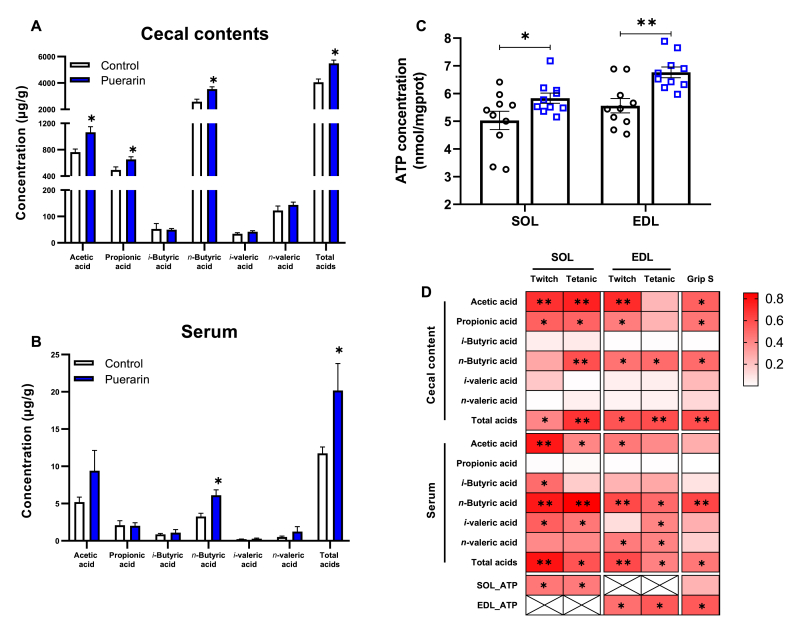
The effects of puerarin in SCFAs and ATP. A& B: Effects of puerarin on the production of SCFAs in cecal contents (A) and serum (B) (N ​= ​5). C: Tissue concentration of ATP in SOL and EDL muscle (N ​= ​10). D: Heatmap shows the value of correlation coefficient among SCFAs, ATP, muscle contraction, and grip strength by Pearson correlation analysis (N ​= ​5). Correlation coefficient R^2^ ​> ​0.3 means moderate correlation and R^2^ ​> ​0.5 means strong correlation, respectively. ∗ and ∗∗ indicate the correlation significance (*P* ​< ​0.05 and *P* ​< ​0.01, respectively). Values shown are the means ​± ​SEM. T-test was used to evaluate differences between the control and the puerarin groups for Fig. 7 A, B and C, ∗*P* ​< ​0.05, and ∗∗*P* ​< ​0.01.

To further explore the effect of puerarin on regulating microbial metabolites, we detected the total SFCAs and its constituted fatty acids concentrations in the cecal contents and serum (Fig. S1). In the cecal contents, the concentrations of acetic acid (39.4%), propionic acid (32.9%), *n*-butyric acid (36.5%), and total SCFAs (35.4%) were significantly higher in the puerarin group than those in the control group (*P* ​< ​0.05) (Fig. 7A). Also in serum, the concentrations of *n*-butyric acid (87.2%) and total SCFAs (71.7%) were significantly higher in the puerarin treatment group compared to those in the control group (*P* ​< ​0.05) (Fig. 7B). ATP concentrations in SOL and EDL muscle were 15.9% (*P* ​< ​0.05) and 21.7% (*P* ​< ​0.01) higher in the puerarin group compared to those in the control group, respectively (Fig. 7C). After treatment with puerarin, the mRNA expressions of GPR41 and GPR43 had no significant difference between the two groups (Fig. S2).

To find possible intrinsic correlations among the SCFAs, ATP, muscle contraction, and grip strength we further analyzed their linear relationships by Pearson's correlation (Fig. 7D, Fig. S3-6). There were significant linear correlations between the concentrations of SCFAs, including cecal total SCFAs, serum *n*-butyric acid and total SCFAs, and skeletal muscle strength and function, including the twitch force and tetanic force of SOL and EDL, as well as the forelimb grip strength (*P* ​< ​0.05) (Fig. 7D, Fig. S3-6). Among these linear correlations, the coefficient of determination was the highest (R^2^ ​= ​0.85) between serum *n*-butyric acid and tetanic force in SOL (Fig. S3). Similarly, there is also a strong linear positive correlation between ATP of EDL and skeletal muscle strength (*P* ​< ​0.05, R^2^ ​> ​0.48) (Fig. S5, Fig. 7D).

## 4 Discussion

The peak muscle mass and strength attained earlier in life may affect the muscle mass and strength in later life. Low muscle strength and poor physical performance in humans were associated with aggravated sarcopenia and increased frailty, fracture risk and mortality [43]. Enhancing the muscle strength attained in early adulthood might be a promising approach to prevent or delay sarcopenia [9]. In this paper, we found that oral administration of puerarin for 8 weeks significantly increased forelimb grip and skeletal muscle contraction in young adult rats (3-month-old). More importantly, it is the first time to reveal the regulatory effect of puerarin left in the intestine [17] on the gut-skeletal muscle axis: the changed gut microbiome augmented SCFA production, including acetic acid, propionic acid and *n*-butyric acid, and potentially increased ATP synthesis and skeletal muscle strength. If gut microbiota is the essential mediator, then intravenous administration of puerarin will not lead to benefits on muscle. Therefore, the intravenous administration should be used in the future to see if gut microbiota is necessary for the muscle improvement.

### 4.1 Puerarin affects gut microbiome distribution in young adult rats

The oral bioavailability of puerarin is very low. In rats, the absolute oral bioavailability of puerarin was 7.50%, 7.29% [17] and 4.21% [44] at doses of 5, 10 and 500 ​mg/kg, respectively. The oral bioavailability of low dose (100 ​mg/kg) was higher than that of high dose (300 ​mg/kg). In this study, the oral dose of puerarin was 150 ​mg/kg, and the bioavailability is speculated no more than 7%, which means approximately 93% remains in the intestine before excretion. Therefore, we speculate that in the microbes-rich intestinal environment, puerarin may interact with intestinal flora, which is likely to be a previously neglected mechanism of puerarin. Recently, gut microbiota was reported to modulate protein, lipid and glucose metabolism, energy and mitochondrial function, and inflammation level in skeletal muscle diseases, including sarcopenia [45]. In addition, there exists a correlation between gut microbiota dysbiosis and decreased physical performance [46]. Therefore, we analyzed the gut microbiota in the cecal content of rats.

The PCoA results demonstrated that the microbial community in the control group varied considerably from that in the puerarin group. We made a careful analysis of different levels of the intestinal flora. At the phylum level, Firmicutes (F) and Bacteroidetes (B) were the dominant phyla detected in the gut microbiota. The ratio of Firmicutes to Bacteroidetes (F/B ratio) was often used as an indicator to measure the health of intestinal flora, and a low F/B ratio may lead to obesity and obesity-related diseases and the high F/B ratio is closely related to inflammatory bowel disease [47]. Our study found that puerarin did not change F/B ratio (*P* ​= ​0.126), suggesting that oral puerarin did not cause intestinal flora dysbiosis.

### 4.2 Puerarin improves skeletal muscle contraction and grip strength mainly by promoting SCFAs and ATP synthesis

SCFAs are a group of volatile saturated fatty acids that contain fewer than six carbon atoms, including acetic acid, propionic acid, butyric acid, valeric acid [19]. Microbial SCFAs are widely proved to mediate the relationship between the gut microbiota and skeletal muscle. The bacterial species *Clostridium butyricum* and *Clostridium symbiosum* and their family Peptococcaceae, from the order Clostridiales, were universal SCFAs-producing bacteria in the gut, especially for butyric acid production [48,49]. Our results showed that puerarin treatment increased the relative abundances of the class Clostridia, and the families Peptococcaceae and Clostridiales, which were identified by LEfSe algorithm as potential microbial markers in the puerarin group compared with that in control group.

SCFAs are the main metabolites produced by the microbiota in the large intestine through the anaerobic fermentation of indigestible polysaccharides such as dietary fiber and resistant starch [19]. Butyrate and propionate formation in the gut occurs mainly from carbohydrate metabolism in glycolysis involving pyruvate (such as TCA cycle), but can also take place from organic acids and amino acids metabolism [50]. In addition, acetate is produced from acetyl-CoA derived from glycolysis and can also be transformed into butyrate by the enzyme butyryl-CoA to acetyl-CoA transferase [51]. Pantothenate is vitamin B5 and is the key precursor for the biosynthesis of coenzyme A (CoA) [52]. Our results revealed that amino acids metabolism, pantothenate and CoA biosynthesis, pyruvate metabolism, TCA cycle, and fatty acid biosynthesis were significantly enhanced in the puerarin group, which might be associated with increased SCFAs biosynthesis. As expected, the cecal and serum n-butyrate and total SCFAs were higher in the puerarin group.

Skeletal muscle oxidative metabolism, glucose uptake, and insulin sensitivity appear to be enhanced by SCFA administration [53]. SCFAs are associated with skeletal muscle mass and strength in pediatric populations [54,55]. Individuals with lower concentration of butyric acid had lower handgrip strength [56]. There were strong linear positive correlations between the concentrations of SCFAs, including cecal total SCFAs, serum *n*-butyric acid and total SCFAs, and skeletal muscle strength and function, including the twitch force and tetanic force of SOL and EDL, as well as the forelimb grip strength. The results are coincided with a metagenomic association analyses, the increased capacity for gut microbial synthesis of the SCFA butyrate was significantly associated with serum butyrate levels and skeletal muscle mass in humans [57].

Several mechanisms of SCFAs improving skeletal muscle strength were proposed [24]. It was reported that feeding a mixture of acetate, propionate, and butyrate in young germ-free mice activated the expression of PGC1α to improve mitochondrial synthesis to inhibit muscle atrophy [58]. Acetate and propionate increase insulin-independent glucose uptake in C2C12 myotubes [53]. Butyrate ameliorates skeletal muscle atrophy in diabetic nephropathy rats by enhancing FFA2-mediated PI3K/Akt/mTOR signals [59]. In addition, as a substrate for ATP synthesis, SCFAs enter the citric acid cycle in the mitochondria to generate ATP and energy for the cells [55]. ATP is the primary energy source and high ATP levels relates to enhancing muscle contraction [60]. Emerging evidence suggests that these bacterial metabolites may act as an important metabolic fuel for skeletal muscle during periods of sustained contraction [24]. We found that puerarin treatment increased the concentrations of ATP in skeletal muscles. In addition, there is also a strong linear positive correlation between serum total SCFAs and ATP, as well as ATP and skeletal muscle strength, including contraction force and forelimb grip strength. Therefore, we speculated that ATP partially involved in the effect of puerarin on promoting skeletal muscle strength.

Besides changing biosynthesis of SCFAs, there had another way by which gut microbiotas affected skeletal muscle tissue [61]. The ratio of two genus bacteria, *Prevotella* (family Prevotellaceae) to *Bacteroides* (family Bacteroidaceae) (P/B ratio) was higher in the sarcopenia group than that in the control group in terms of relative abundance. Therefore, the high ratio of P/B was used to predict sarcopenia in human [61]. We found that puerarin significantly decreased P/B ratio in the intestinal flora, which indicated the possibility that puerarin might be beneficial against sarcopenia in a way of SCFAs.

So far, both of exercise and nutrition remain the cheapest and most effective means of preventing or treating sarcopenia. Exercise and nutrition treatment have been the most common used intervention among elderly populations and showed overall significant positive effects on muscle strength and physical performance [62,63]. In the future, puerarin alone or combined with exercise should be designed and explored.

The strategy “enhancing muscle mass and strength in young adulthood might be a promising strategy to prevent or delay sarcopenia in later life” has not been well established and accepted [3,9]. The long-term impact of beneficial effects on skeletal muscle in young should be further investigated. Puerarin improves skeletal muscle strength by regulating gut microbiota in young adult rats. Would it work in aged? That might be a next step. The effectiveness of puerarin plays in preventing or treating sarcopenia is waiting for us to continue.

## 5 Conclusions

In conclusion, puerarin improved the grip strength and muscle contraction function in young adult rats. The underlying mechanism may include that puerarin increased SCFAs production by regulating gut microbiota, and augmented ATP synthesis in skeletal muscle.

## Funding

This work was supported by the 10.13039/501100001809National Nature Science Foundation of China (81773964), the 10.13039/501100017610Shenzhen Science and Technology Innovation Committee (JCYJ20210324102006017) and Joint Laboratory for Biomaterials SIAT–HKU–CUHK under CUHK-10.13039/501100002367CAS Joint Laboratory Fund (Project Code: 4750376).

## Author contribution

Xinluan Wang and Wenyao Yang designed the research. Wenyao Yang carried out the experiments and performed data analysis. Bimin Gao did data analysis of microbiomics. Wenyao Yang, Bimin Gao and Xinluan Wang wrote the manuscript. Xinluan Wang and Ling Qin revised the manuscript. All authors have read and approved the final manuscript. Approval of the version of the manuscript to be published (the names of all authors must be listed): W.Y. Yang, B.M. Gao, L. Qin, X.L. Wang.

## Declaration of competing interest

The authors have no conflicts of interest to disclose about this article.
