# Supplementary material for: Puerarin improves skeletal muscle strength by regulating gut microbiota in young adult rats
Source: J Orthop Translat. 2022 Sep 21;35:87–98. doi: 10.1016/j.jot.2022.08.009 (PMC9508383; doi:10.1016/j.jot.2022.08.009)
Supplement: Multimedia component 1 [file mmc1.pptx]

## Slide 1
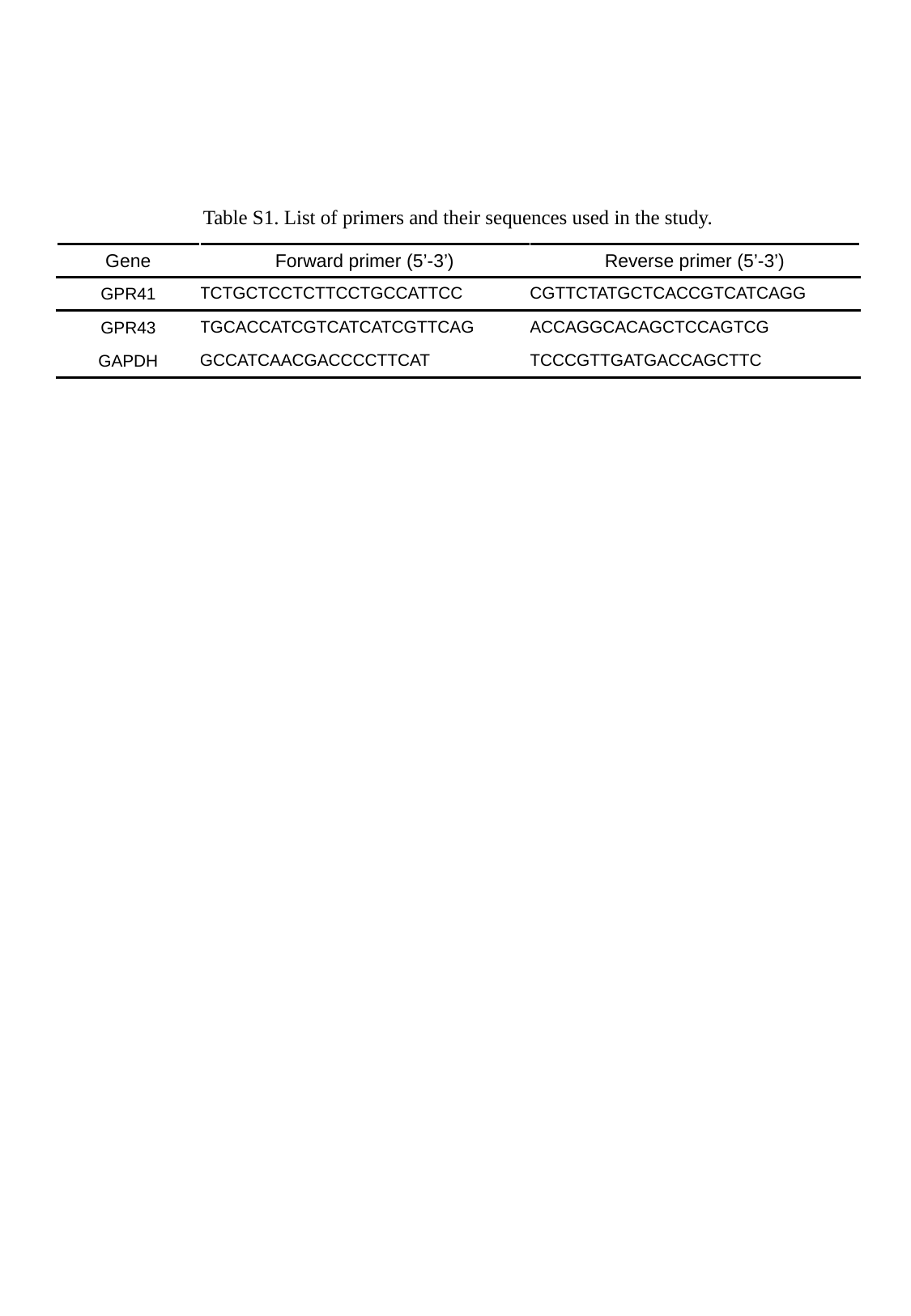

Table S1. List of primers and their sequences used in the study.
| Gene | Forward primer (5’-3’) | Reverse primer (5’-3’) |
| --- | --- | --- |
| GPR41 | TCTGCTCCTCTTCCTGCCATTCC | CGTTCTATGCTCACCGTCATCAGG |
| GPR43 | TGCACCATCGTCATCATCGTTCAG | ACCAGGCACAGCTCCAGTCG |
| GAPDH | GCCATCAACGACCCCTTCAT | TCCCGTTGATGACCAGCTTC |

## Slide 2
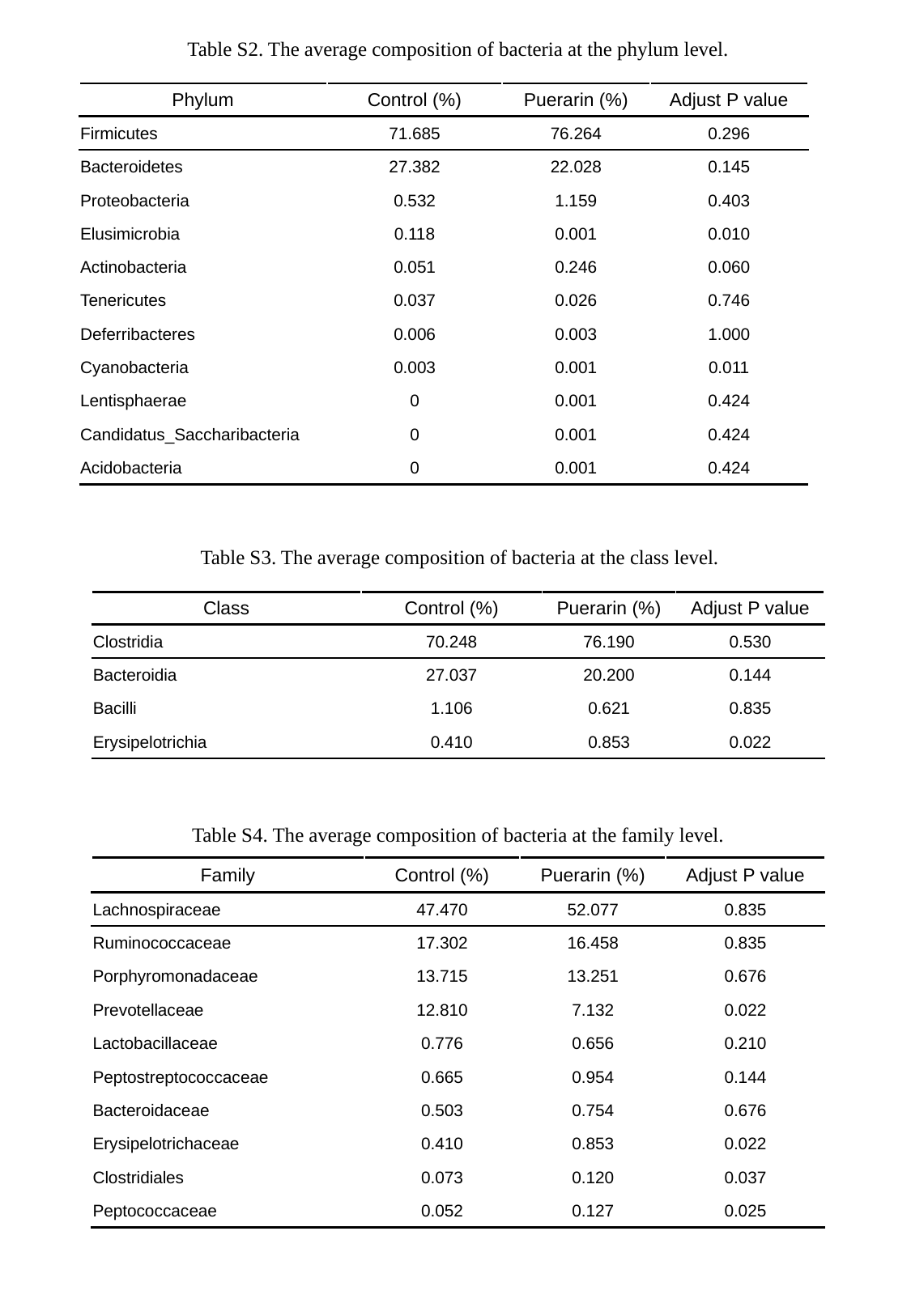

Table S2. The average composition of bacteria at the phylum level.
| Phylum | Control (%) | Puerarin (%) | Adjust P value |
| --- | --- | --- | --- |
| Firmicutes | 71.685 | 76.264 | 0.296 |
| Bacteroidetes | 27.382 | 22.028 | 0.145 |
| Proteobacteria | 0.532 | 1.159 | 0.403 |
| Elusimicrobia | 0.118 | 0.001 | 0.010 |
| Actinobacteria | 0.051 | 0.246 | 0.060 |
| Tenericutes | 0.037 | 0.026 | 0.746 |
| Deferribacteres | 0.006 | 0.003 | 1.000 |
| Cyanobacteria | 0.003 | 0.001 | 0.011 |
| Lentisphaerae | 0 | 0.001 | 0.424 |
| Candidatus\_Saccharibacteria | 0 | 0.001 | 0.424 |
| Acidobacteria | 0 | 0.001 | 0.424 |
Table S3. The average composition of bacteria at the class level.
| Class | Control (%) | Puerarin (%) | Adjust P value |
| --- | --- | --- | --- |
| Clostridia | 70.248 | 76.190 | 0.530 |
| Bacteroidia | 27.037 | 20.200 | 0.144 |
| Bacilli | 1.106 | 0.621 | 0.835 |
| Erysipelotrichia | 0.410 | 0.853 | 0.022 |
Table S4. The average composition of bacteria at the family level.
| Family | Control (%) | Puerarin (%) | Adjust P value |
| --- | --- | --- | --- |
| Lachnospiraceae | 47.470 | 52.077 | 0.835 |
| Ruminococcaceae | 17.302 | 16.458 | 0.835 |
| Porphyromonadaceae | 13.715 | 13.251 | 0.676 |
| Prevotellaceae | 12.810 | 7.132 | 0.022 |
| Lactobacillaceae | 0.776 | 0.656 | 0.210 |
| Peptostreptococcaceae | 0.665 | 0.954 | 0.144 |
| Bacteroidaceae | 0.503 | 0.754 | 0.676 |
| Erysipelotrichaceae | 0.410 | 0.853 | 0.022 |
| Clostridiales | 0.073 | 0.120 | 0.037 |
| Peptococcaceae | 0.052 | 0.127 | 0.025 |

## Slide 3
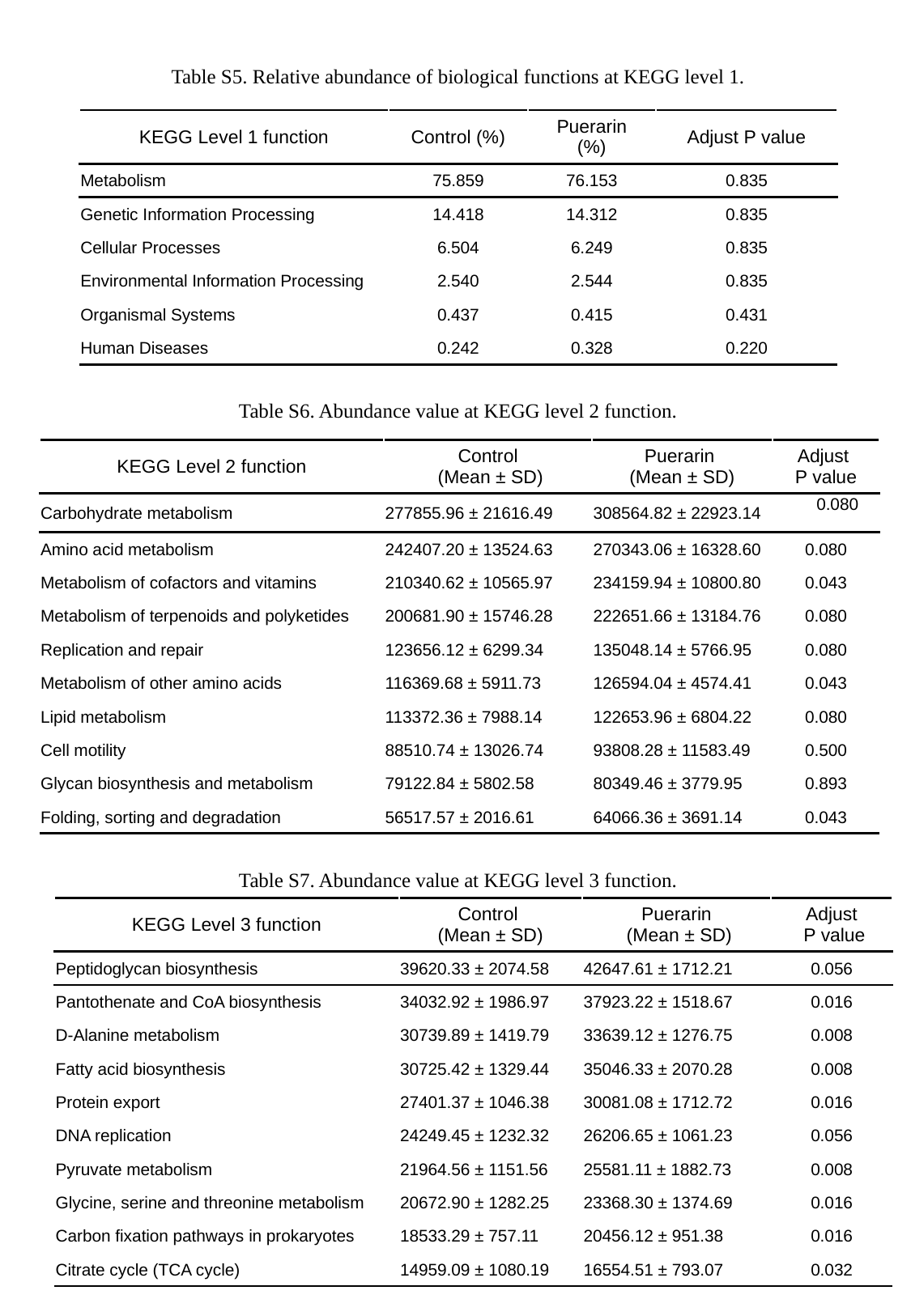

Table S5. Relative abundance of biological functions at KEGG level 1.
| KEGG Level 1 function | Control (%) | Puerarin (%) | Adjust P value |
| --- | --- | --- | --- |
| Metabolism | 75.859 | 76.153 | 0.835 |
| Genetic Information Processing | 14.418 | 14.312 | 0.835 |
| Cellular Processes | 6.504 | 6.249 | 0.835 |
| Environmental Information Processing | 2.540 | 2.544 | 0.835 |
| Organismal Systems | 0.437 | 0.415 | 0.431 |
| Human Diseases | 0.242 | 0.328 | 0.220 |
Table S6. Abundance value at KEGG level 2 function.
| KEGG Level 2 function | Control (Mean ± SD) | Puerarin (Mean ± SD) | Adjust P value |
| --- | --- | --- | --- |
| Carbohydrate metabolism | 277855.96 ± 21616.49 | 308564.82 ± 22923.14 | 0.080 |
| Amino acid metabolism | 242407.20 ± 13524.63 | 270343.06 ± 16328.60 | 0.080 |
| Metabolism of cofactors and vitamins | 210340.62 ± 10565.97 | 234159.94 ± 10800.80 | 0.043 |
| Metabolism of terpenoids and polyketides | 200681.90 ± 15746.28 | 222651.66 ± 13184.76 | 0.080 |
| Replication and repair | 123656.12 ± 6299.34 | 135048.14 ± 5766.95 | 0.080 |
| Metabolism of other amino acids | 116369.68 ± 5911.73 | 126594.04 ± 4574.41 | 0.043 |
| Lipid metabolism | 113372.36 ± 7988.14 | 122653.96 ± 6804.22 | 0.080 |
| Cell motility | 88510.74 ± 13026.74 | 93808.28 ± 11583.49 | 0.500 |
| Glycan biosynthesis and metabolism | 79122.84 ± 5802.58 | 80349.46 ± 3779.95 | 0.893 |
| Folding, sorting and degradation | 56517.57 ± 2016.61 | 64066.36 ± 3691.14 | 0.043 |
Table S7. Abundance value at KEGG level 3 function.
| KEGG Level 3 function | Control (Mean ± SD) | Puerarin (Mean ± SD) | Adjust P value |
| --- | --- | --- | --- |
| Peptidoglycan biosynthesis | 39620.33 ± 2074.58 | 42647.61 ± 1712.21 | 0.056 |
| Pantothenate and CoA biosynthesis | 34032.92 ± 1986.97 | 37923.22 ± 1518.67 | 0.016 |
| D-Alanine metabolism | 30739.89 ± 1419.79 | 33639.12 ± 1276.75 | 0.008 |
| Fatty acid biosynthesis | 30725.42 ± 1329.44 | 35046.33 ± 2070.28 | 0.008 |
| Protein export | 27401.37 ± 1046.38 | 30081.08 ± 1712.72 | 0.016 |
| DNA replication | 24249.45 ± 1232.32 | 26206.65 ± 1061.23 | 0.056 |
| Pyruvate metabolism | 21964.56 ± 1151.56 | 25581.11 ± 1882.73 | 0.008 |
| Glycine, serine and threonine metabolism | 20672.90 ± 1282.25 | 23368.30 ± 1374.69 | 0.016 |
| Carbon fixation pathways in prokaryotes | 18533.29 ± 757.11 | 20456.12 ± 951.38 | 0.016 |
| Citrate cycle (TCA cycle) | 14959.09 ± 1080.19 | 16554.51 ± 793.07 | 0.032 |

## Slide 4
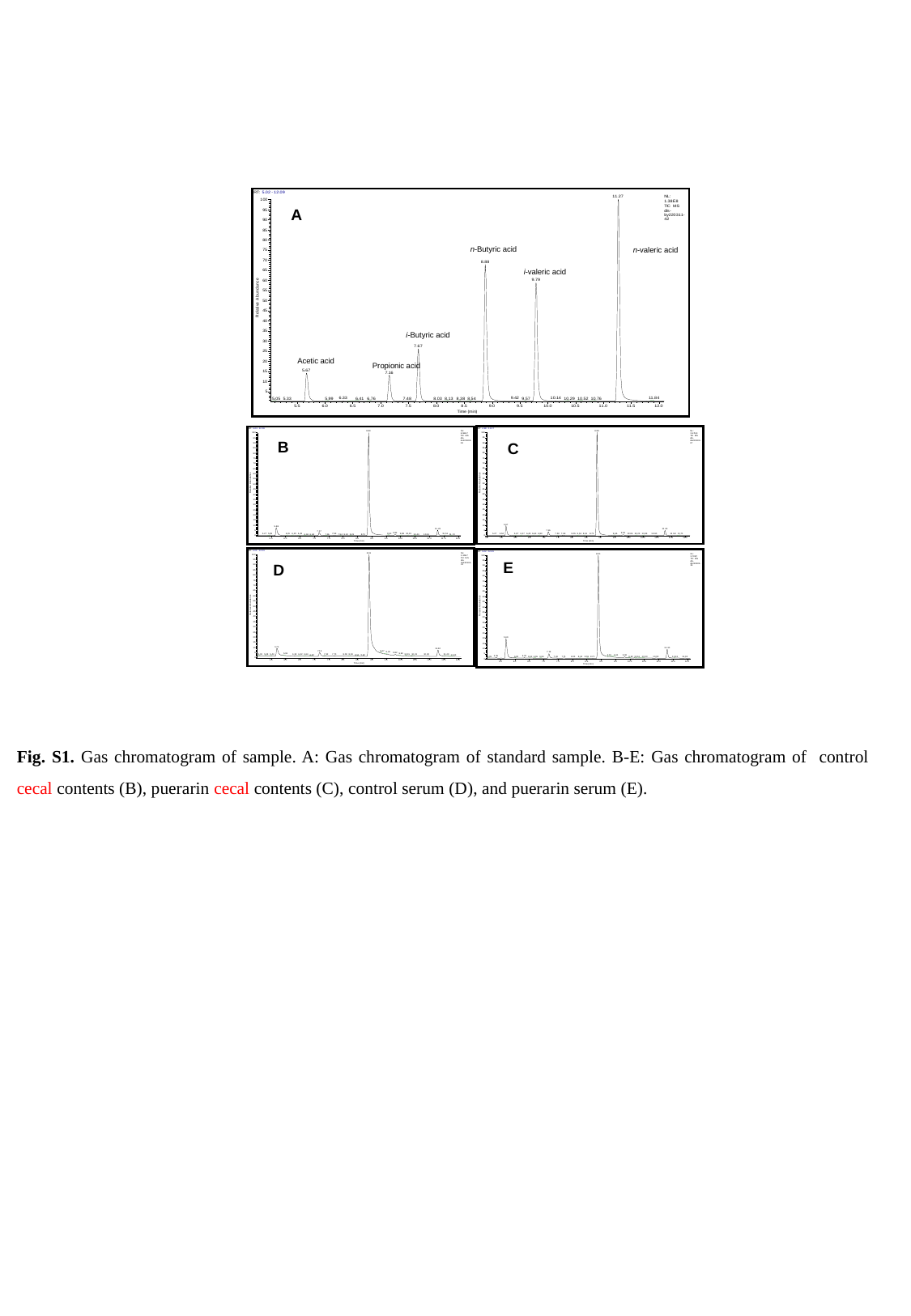

n-Butyric acid
n-valeric acid
i-valeric acid
i-Butyric acid
Acetic acid
Propionic acid
A
B
C
E
D
Fig. S1. Gas chromatogram of sample. A: Gas chromatogram of standard sample. B-E: Gas chromatogram of control cecal contents (B), puerarin cecal contents (C), control serum (D), and puerarin serum (E).

## Slide 5
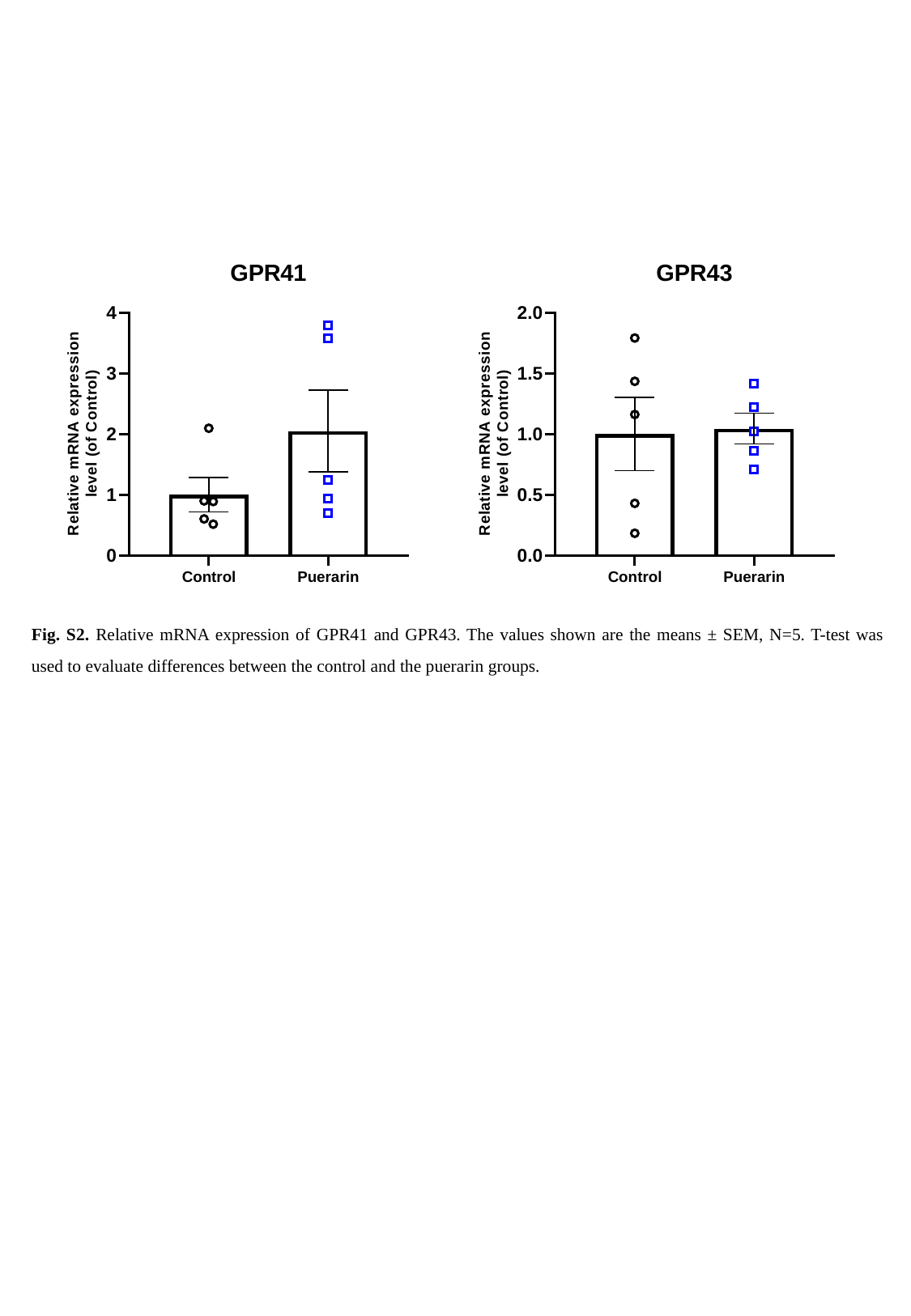

Fig. S2. Relative mRNA expression of GPR41 and GPR43. The values shown are the means ± SEM, N=5. T-test was used to evaluate differences between the control and the puerarin groups.

## Slide 6
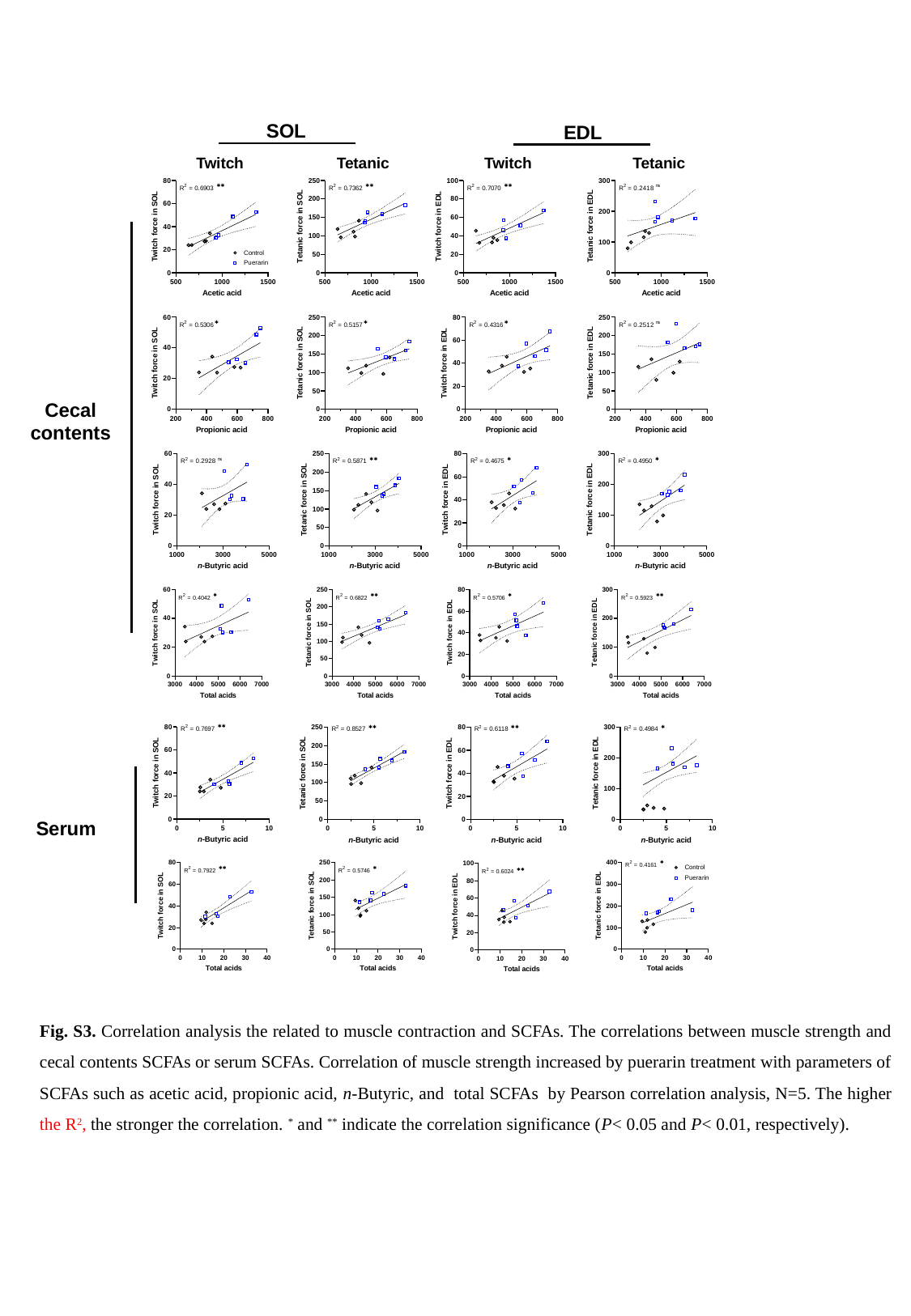

SOL
EDL
Twitch
Tetanic
Twitch
Tetanic
Cecal
contents
Serum
Fig. S3. Correlation analysis the related to muscle contraction and SCFAs. The correlations between muscle strength and cecal contents SCFAs or serum SCFAs. Correlation of muscle strength increased by puerarin treatment with parameters of SCFAs such as acetic acid, propionic acid, n-Butyric, and total SCFAs by Pearson correlation analysis, N=5. The higher the R2, the stronger the correlation. * and ** indicate the correlation significance (P< 0.05 and P< 0.01, respectively).

## Slide 7
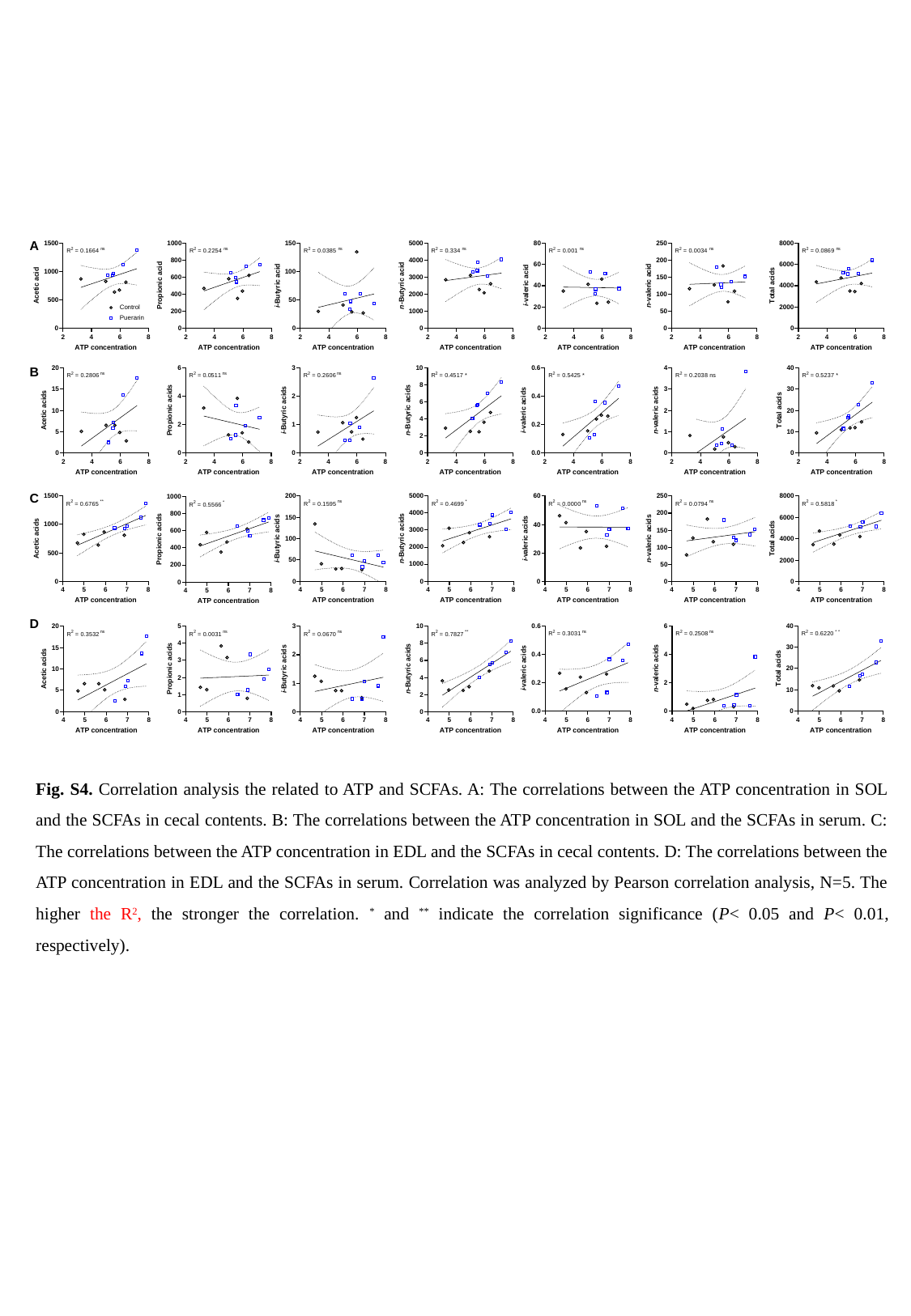

A
B
C
D
Fig. S4. Correlation analysis the related to ATP and SCFAs. A: The correlations between the ATP concentration in SOL and the SCFAs in cecal contents. B: The correlations between the ATP concentration in SOL and the SCFAs in serum. C: The correlations between the ATP concentration in EDL and the SCFAs in cecal contents. D: The correlations between the ATP concentration in EDL and the SCFAs in serum. Correlation was analyzed by Pearson correlation analysis, N=5. The higher the R2, the stronger the correlation. * and ** indicate the correlation significance (P< 0.05 and P< 0.01, respectively).

## Slide 8
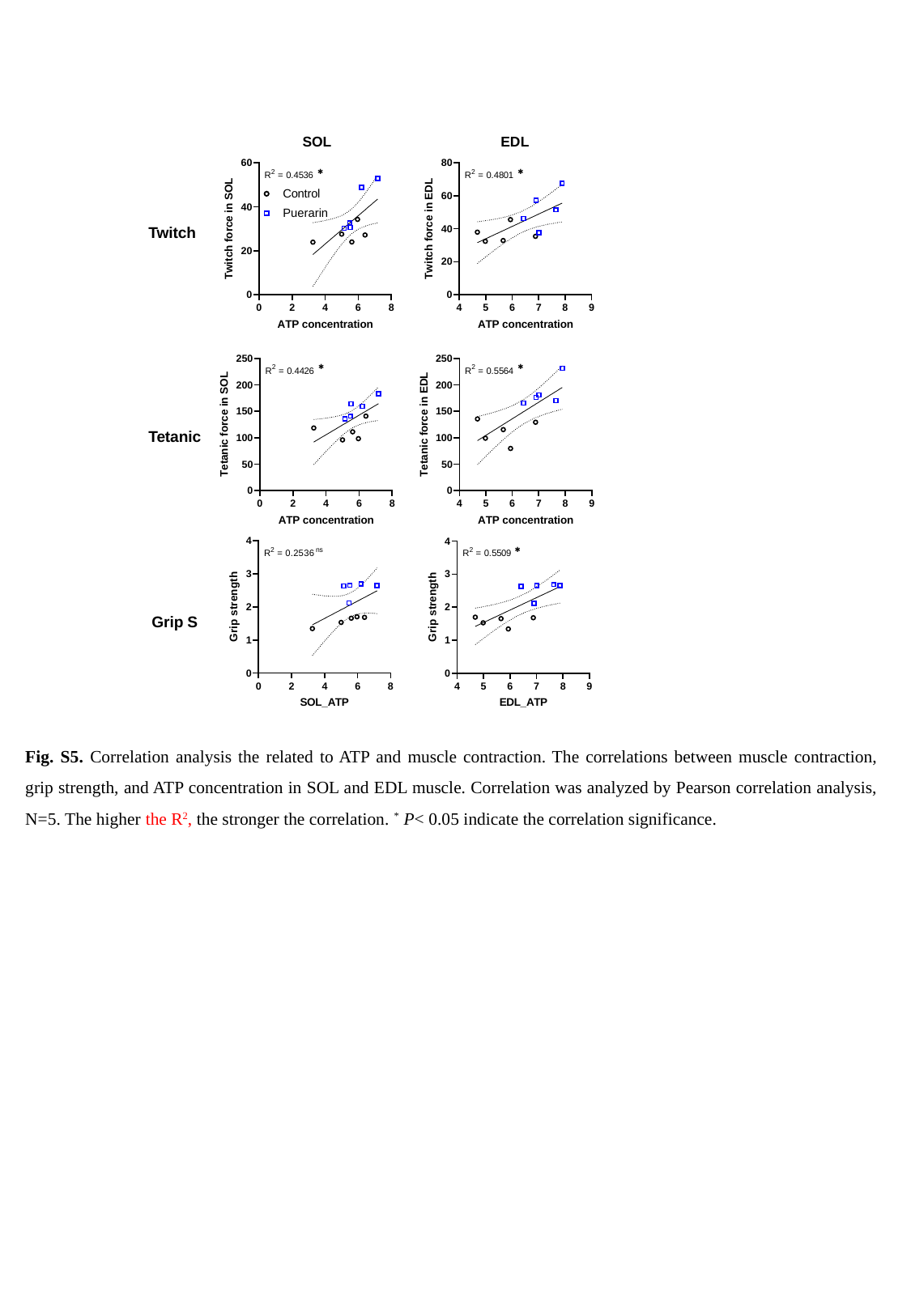

SOL
EDL
Twitch
Tetanic
Grip S
Fig. S5. Correlation analysis the related to ATP and muscle contraction. The correlations between muscle contraction, grip strength, and ATP concentration in SOL and EDL muscle. Correlation was analyzed by Pearson correlation analysis, N=5. The higher the R2, the stronger the correlation. * P< 0.05 indicate the correlation significance.

## Slide 9
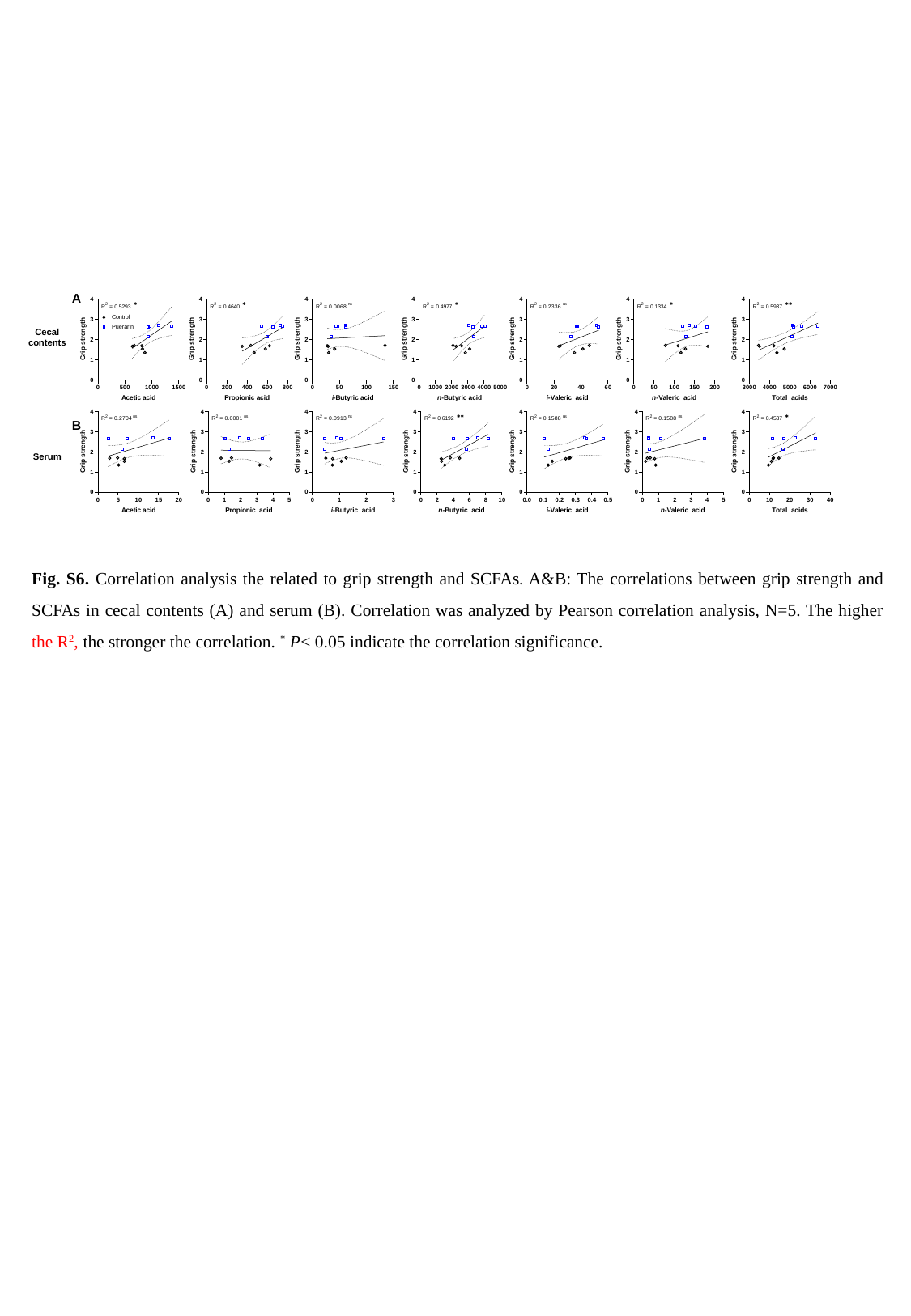

A
Cecal
contents
B
Serum
Fig. S6. Correlation analysis the related to grip strength and SCFAs. A&B: The correlations between grip strength and SCFAs in cecal contents (A) and serum (B). Correlation was analyzed by Pearson correlation analysis, N=5. The higher the R2, the stronger the correlation. * P< 0.05 indicate the correlation significance.
